# The effect of ambulatory blood pressure load on mitral regurgitation in continuous ambulatory peritoneal dialysis patients

**DOI:** 10.1515/med-2025-1155

**Published:** 2025-04-01

**Authors:** Qingyun Li, Xinqiang Zhong, Tongxia Cui, Bairong Chen, Weiping Zhu

**Affiliations:** Department of Nephrology, The Fifth Affiliated Hospital of Sun Yat-Sen University, Zhuhai, Guang Dong Province, China; Department of Cardiology, The Fifth Affiliated Hospital of Sun Yat-sen University, Zhuhai, Guang Dong Province, China

**Keywords:** ambulatory blood pressure load, cardiovascular disease, mitral regurgitation area, continuous ambulatory peritoneal dialysis

## Abstract

**Background:**

Hypertension is a risk factor for cardiovascular disease. The present study aimed to explore the impact of ambulatory blood pressure load (BPL) on mitral regurgitation (MR) in continuous ambulatory peritoneal dialysis (CAPD) patients.

**Methods:**

A total of 215 CAPD patients hospitalized in the Department of Nephrology at the Fifth Affiliated Hospital of Sun Yat-sen University between November 2017 and June 2022 were included in the study. All subjects underwent 24-h ambulatory BP monitoring and an echocardiography examination. BPL and MR area (MRA) were calculated. Subjects were divided into high regurgitation group and non-high regurgitation group. General data were also collected. The effect of ambulatory BPL on MR in CAPD patients was analyzed using multiple linear regression.

**Results:**

Baseline data comparison revealed statistically significant differences in hemoglobin, serum β_2_-microglobulin, NT-proBNP, left atrial diameter (LAD), left ventricular end-diastolic diameter (LVDD), LV ejection fraction (LVEF), LV fractional shortening (LVFS), and mitral valve calcification between the high and non-high regurgitation groups (*P* < 0.05). There was no statistically significant difference in other clinical characteristics. Correlation analysis showed correlations between MRA and 24-h systolic BPL (24h-SBPL), 24-h diastolic BPL (24h-DBPL), daytime systolic BPL (D-SBPL), daytime DBPL (D-DBPL), nighttime systolic BPL (N-SBPL), nighttime DBPL (N-DBPL), hemoglobin level, NT-proBNP level, LAD, LVDD, LVEF, and LVFS. Multiple linear regression analysis revealed that the effects of 24h-DBPL and D-DBPL on MRA were statistically significantly different (*P* < 0.05).

**Conclusions:**

Ambulatory BPL, particularly 24h-DBPL and D-DBPL, significantly affected MR. Therefore, controlling BPL, especially DBPL, was essential for reducing the incidence of cardiovascular events and clinical mortality in CAPD patients.

## 1 Introduction

Chronic kidney disease (CKD) refers to the structural or functional abnormalities of the kidney caused by various factors for more than 3 months. According to the guidelines formulated by the Kidney Disease Outcome Quality Initiative (K/DOQI), CKD is categorized into stages 1–5, where stage 5 is also known as end-stage renal disease (ESRD) [1]. Continuous ambulatory peritoneal dialysis (CAPD) is one of the most common forms of renal replacement therapy used for ESRD patients, which greatly reduces their mortality. However, cardiovascular diseases (CVDs) remain the primary cause of death in ESRD patients on CAPD [2], accounting for 50% of total mortality in dialysis patients [3]. Heart failure is the most common CVD in the CKD population. The Acute Dialysis Quality Initiative XI Working Group has proposed eight echocardiographic criteria for heart failure in ESRD dialysis patients, including left ventricular (LV) hypertrophy, LV ejection fraction (LVEF) of ≤45%, increased LV volume index, right ventricular systolic dysfunction, left atrial enlargement, diastolic dysfunction, mitral or aortic valve disease, and segmental wall motion abnormality [4]. LV hypertrophy, cardiac enlargement, systolic dysfunction, diastolic dysfunction, and heart valve regurgitation are generally recognized as the main characteristics of CVD in ESRD patients [5,6]. Hypertension is a significant traditional risk factor for CVD in CAPD patients. Therefore, effective blood pressure (BP) management can mitigate the risk of CVD in this population. Given the limitations of office-measured BP, the 2021 Kidney Disease: Improving Global Outcomes (KDIGO) clinical practice guideline recommends supplementing standardized office BP measurement with ambulatory BP monitoring (ABPM) in hypertension management [7]. Because BP values obtained from 24-h ABPM strongly correlate with cardiovascular and renal outcomes, it is considered the preferred method for measuring BP in the general population and CKD patients [8]. As a consequence, ambulatory BP load (BPL) measured using 24-h ABPM has been paid more and more attention in clinical practice. The aim of the present study was to investigate the effect of ambulatory BPL on mitral regurgitation (MR) in maintenance peritoneal dialysis (PD) patients by means of 24-h ABPM monitoring and cardiac color Doppler ultrasound in order to reduce the incidence of cardiovascular events and clinical mortality, thereby improving patient prognosis.

## 2 Materials and methods

### 2.1 Study design and participants

This investigation was a retrospective study of 215 CAPD patients who were hospitalized in the Department of Nephrology at the Fifth Affiliated Hospital of Sun Yat-sen University between November 2017 and June 2022.

Inclusion Criteria:

- Age of 18 years or older;
- Dialysis duration of at least three months;
- No infection, severe heart failure, or coronary artery or cerebrovascular events in the past month.

Exclusion Criteria:

- Patients on PD combined with hemodialysis;
- Patients with congenital heart disease, hypertrophic cardiomyopathy, or valvular heart disease;
- Patients who had undergone cardiac valve replacement;
- Patients who could not tolerate ABPM;
- Pregnant and lactating patients; and
- Patients unable to care for themselves or those suffering from mental illness and therefore unable to cooperate with the study procedure.

### 2.2 Research methods

#### 2.2.1 MR area (MRA) calculation and grouping

**Figure 1:**
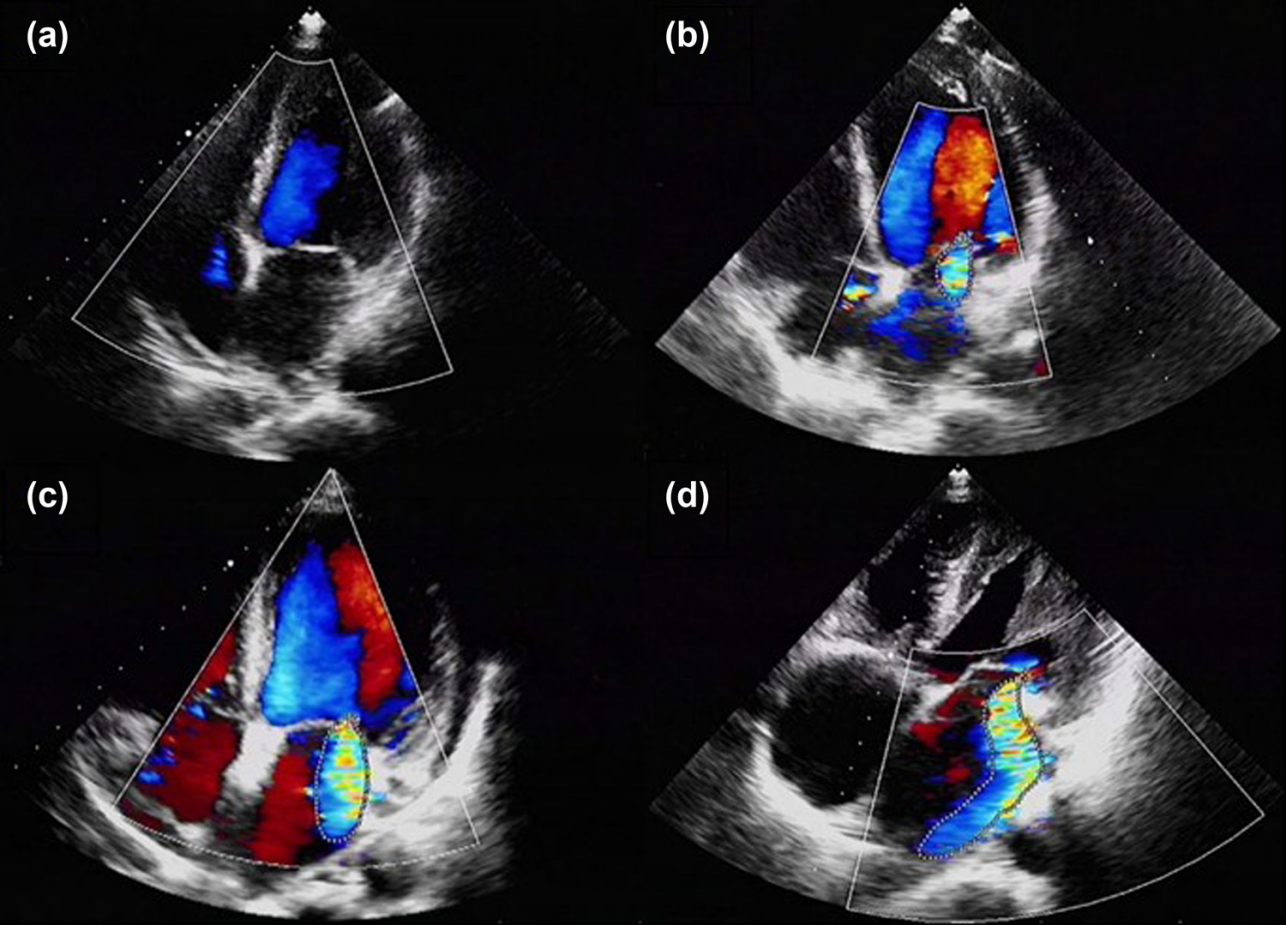
Schematic diagram of MR. Blue, green, or mosaic signal spreading from the mitral valve to the left atrium during ventricular systole represents the extent of mitral regurgitation area (highlighted by dashed lines). Schematic diagram of (a) mitral valve without regurgitation, (b) mild mitral regurgitation, (c) moderate mitral regurgitation, and (d) severe mitral regurgitation.

Color Doppler ultrasound (Philips, Bothell, WA, USA) was used by the same professional cardiac sonographer to perform two-dimensional cardiac color ultrasound examination on the enrolled PD patients. The main observations included mitral valve structural changes, valve regurgitation, and valve calcification. Cardiac MR was considered to be present if a blue, green, or mosaic signal was found to spread from the mitral valve to the left atrium during LV contraction (Figure 1) [9]. The maximum systolic MR image was obtained by continuously adjusting the orientation of the probe. Subsequently, MRA was calculated and averaged by two clinicians using Image J image processing software(Developed by the National Institutes of Health). The degree of MR was classified into mild (0 cm^2^ ≤ MRA < 4 cm^2^), moderate (4 cm^2^ ≤ MRA ≤ 8 cm^2^), and severe (MRA > 8 cm^2^) based on the guidelines provided in the ninth edition of Internal Medicine published by the People’s Medical Publishing House. Patients with moderate regurgitation and above were categorized into the high regurgitation group, while those with mild or no regurgitation were placed into the non-high regurgitation group.

#### 2.2.2 ABPM

Patients underwent 24-h ABPM using a Mobil-O-Graph^®^ Ambulatory BP recording analyzer (I.E.M, Stolberg, Germany). The appropriate cuff size was selected according to the patient’s arm circumference, and the cuff was fixed on the non-dominant arm (usually on the left upper arm). BP was measured every 20 min during the day (from 6 am to 11 pm) and every 30 min at night (from 11 pm to 6 am). At the same time, BP readings were analyzed by the hypertension management software (HMS CS, Version 4.8) providing data on BPL, pulse pressure, and other parameters. BPL refers to the proportion of BP measurements above the normal value to the total number of BP measurements [10]. The reference standards for ABPM on the ambulatory BP recording analyzer used in the present study were as follows. Daytime hypertension was defined as systolic blood pressure ≥140 mmHg and/or diastolic blood pressure ≥90 mmHg, and nocturnal hypertension as systolic blood pressure ≥125 mmHg and/or diastolic blood pressure ≥80 mmHg. On the day of monitoring, patients were allowed to carry out their daily activities while avoiding strenuous physical exertion and were asked to remain still during BP measurements. Minor trauma, such as blood collection, was avoided to prevent congestion or infection.

#### 2.2.3 Statistical analysis

Statistical analysis was carried out using SPSS Statistics 25.0 (Developed by International Business Machines Corporation). All quantitative data were tested for normality. Normally distributed data were expressed as mean ± standard deviation ( \[\bar{x}]\] ± s) and as median otherwise (interquartile range). Several tests were employed for the comparison of quantitative data for two independent samples. Independent sample *t*-test was used if both samples met the normal distribution and variance homogeneity requirements. Corrected *t*-test was used if the samples satisfied the normal distribution requirement but their variance was not homogeneous. Mann–Whitney *U* nonparametric test was used if a sample did not satisfy the normal distribution criteria. Categorical data were expressed as percentages and comparisons between groups was performed using the chi-square test. Pearson’s correlation analysis was used for parameters with normal distribution, and Spearman correlation analysis was used for parameters with non-normal distribution. Variables with statistical significance in correlation analysis were further evaluated using multiple linear regression analysis. *P* < 0.05 was considered statistically significant.


**Ethics:** This project is a retrospective study and therefore an informed waiver has been passed. The present study have been performed with consent in compliance with the Helsinki Declaration and was approved by the Ethics Committee of the Fifth Affiliated Hospital of Sun Yat-sen University (Ethics No. K209-1).

## 3 Results

### 3.1 Clinical characteristics of study subjects

Baseline data for 215 CAPD patients included in the study showed statistically significant differences in hemoglobin level, β_2_-microglobulin level, NT-proBNP level, left atrial diameter (LAD), LV end-diastolic diameter (LVDD), LVEF, LV fractional shortening (LVFS), and mitral valve calcification between the high and non-high regurgitation groups (Table 1). No statistical difference was observed in other clinical characteristics.

**Table 1:** Clinical characteristics of study subjects

Subjects	High regurgitation group	Non-high regurgitation group	*P* value	*t* value/*Z* value/*X* ^2^ value
Number of cases	34	181		
Gender (male/female)	11/23	82/99	0.162	1.956
Age (years)	48.50 (45.00,57.25)	47.00 (39.00,57.00)	0.182	−1.336
Body mass index (BMI) (kg/m^2^)	24.33 ± 3.71	23.22 (21.32,25.98)	0.567	−0.572
Peritoneal dialysis months (months)	43.00 (12.50,81.25)	28.00 (12.00,62.50)	0.482	−0.703
Hemoglobin (g/L)	97.38 ± 15.34	105.42 ± 19.33	0.010*	−2.589
24h-SBPL (%)	70.00 (44.25,95.25)	61.00 (28.00,84.00)	0.098	−1.656
24h-DBPL (%)	80.50 (54.75,92.00)	73.00 (42.00,90.00)	0.195	−1.297
D-SBPL (%)	62.50 (35.00,94.00)	52.00 (15.50,80.00)	0.071	−1.804
D-DBPL (%)	78.50 (45.50,89.00)	67.00 (36.00,88.00)	0.186	−1.322
N-SBPL (%)	100.00 (62.25,100.00)	92.00 (62.00,100.00)	0.289	−1.060
N-DBPL (%)	97.00 (83.75,100.00)	92.00 (63.00,100.00)	0.137	−1.487
Parathyroid hormone (ρmol/L)	31.05 (16.63,54.15)	37.49 (24.52,62.89)	0.141	−1.472
Blood phosphorus (mmol/L)	1.66 (1.43,2.06)	1.70 (1.39,2.07)	0.917	−0.104
Blood calcium (mmol/L)	2.08 (1.94,2.27)	2.14 (2.04,2.29)	0.210	−1.255
serum β_2_-microglobulin (mg/L)	34.15 (27.43,45.64)	32.93 (23.26,38.26)	0.038*	−2.075
Serum albumin (g/L)	32.60 (30.03,34.13)	33.50 (31.20,36.20)	0.091	−1.692
Total cholesterol (mmol/L)	4.25 (3.67,4.94)	4.23 (3.40,5.14)	0.793	−0.263
Low-density lipoprotein cholesterol (mmol/L)	2.33 (1.60,2.71)	2.27 (1.68,2.87)	0.917	−0.104
25-OH vitamin D (ng/mL)	11.32 (8.17,16.18)	12.43 (9.42,17.54)	0.196	−1.292
24-h Pulse pressure (mmHg)	46.00 (59.00,40.00)	45.00 (39.00,53.50)	0.300	−1.036
NT-proBNP (pg/ml)	25850.00 (5797.50,35000.00)	3480.00 (1160.00,10950.00)	<0.001**	−5.051
LAD (mm)	41.50 (37.00,45.00)	37.00 (33.00,40.00)	<0.001**	−4.496
LVDD (mm)	53.00 (48.75,57.25)	49.00 (45.50,53.00)	0.003**	−3.013
LVEF (%)	65.50 (60.25，68.25)	69.00 (64.00,73.00)	0.003**	−3.013
LVFS (%)	36.00 (32.50,39.25)	38.00 (34.50,42.00)	0.008**	−2.637
Coronary artery calcium score (score)	150.40 (18.28,1563.58)	77.70 (0.00,660.39)	0.115	−1.577
Diabetes mellitus (yes/no)	10/24	28/153	0.051	3.824
Mitral valve calcification (yes/no)	12/22	24/157	0.002**	9.969

Data are expressed as median (interquartile range) or mean ± SD, * denotes *P* < 0.05, ** denotes *P* < 0.01.

### 3.2 Correlation analysis between each subject and MRA

According to Spearman’s correlation analysis results, no correlation was observed among β_2_-microglobulin, mitral valve calcification, and MRA. However, weak correlations were present among 24-h SBP load (24h-SBPL), 24-h diastolic BP load (24h-DBPL), daytime SBPL (D-SBPL), daytime DBPL (D-DBPL), nighttime SBPL (N-SBPL), nighttime DBPL (N-DBPL), hemoglobin level, NT-proBNP level, LAD, LVDD, LVEF, LVFS, and MRA (Table 2).

**Table 2:** Spearman’s correlation analysis between each subject and MRA

	*r* Value	*P* value
24-h SBPL (%)	0.281	<0.001**
24-h DBPL (%)	0.246	<0.001**
D-SBPL (%)	0.294	<0.001**
D-DBPL (%)	0.248	<0.001**
N-SBPL (%)	0.210	0.002**
N-DBPL (%)	0.228	0.001**
Hemoglobin (g/L)	−0.176	0.010*
Serum β_2_-microglobulin (mg/L)	0.124	0.070
NT-proBNP	0.429	<0.001**
LAD (mm)	0.361	<0.001**
LVDD (mm)	0.281	<0.001**
LVEF (%)	−0.197	0.004**
LVFS (%)	−0.197	0.004**
Mitral valve calcification (yes/no)	0.133	0.051

Note: * denotes *P* < 0.05, ** denotes *P* < 0.01.

### 3.3 Multiple linear regression analysis of BPL and other variables with MRA

Multiple linear regression models were constructed using BPL, age, duration (months) of peritoneal dialysis, body mass index (BMI), total cholesterol level, LAD, LVDD, LVEF, NT-proBNP level, mitral valve calcification, 24-h pulse pressure, and MRA. The results showed that the effect of 24h-DBPL on MRA was statistically significant (*B* = 0.010, *t* = 2.078, *P* = 0.039). In addition, the effect of D-DBPL on MRA was statistically different (*B* = 0.010, *t* = 2.106, *P* = 0.036; Tables 3–8).

**Table 3:** Multiple linear regression analysis of 24-h SBPL and other variables with MRA

Variables	*β*	*t*	*P*
24-h SBPL	0.091	0.965	0.335
Age	0.165	2.182	0.030*
Peritoneal dialysis months	−0.122	−1.880	0.062
BMI	−0.034	−0.482	0.631
Total cholesterol	−0.006	−0.096	0.924
LAD	0.190	1.764	0.079
LVDD	−0.017	−0.163	0.871
LVEF	−0.074	−0.911	0.363
NT-proBNP	0.339	4.129	<0.001*
Mitral valve calcification	0.132	1.925	0.056
24-h pulse pressure	−0.084	−0.834	0.405
The adjusted *R* ^2^	0.248		

Note: SE, standard error; VIF, variance inflation factor; *R* ^2^, coefficient of determination; * denotes *P* < 0.05.

**Table 4:** Multiple linear regression analysis of 24-h DBPL and other variables with MRA

Variables	*β*	*t*	*P*
24-h DBPL	0.142	2.078	0.039*
Age	0.191	2.536	0.012*
Peritoneal dialysis months	−0.123	−1.908	0.058
BMI	−0.029	−0.410	0.682
Total cholesterol	−0.012	−0.187	0.852
LAD	0.178	1.662	0.098
LVDD	−0.030	−0.297	0.767
LVEF	−0.082	−1.015	0.312
NT-proBNP	0.334	4.110	0.000*
Mitral valve calcification	0.139	2.044	0.042*
24-h pulse pressure	−0.050	−0.663	0.508
The adjusted *R* ^2^	0.260		

Note: SE, standard error; VIF, variance inflation factor; *R* ^2^, coefficient of determination; * denotes *P* < 0.05.

**Table 5:** Multiple linear regression analysis of D-SBPL and other variables with MRA

Variables	*β*	*t*	*P*
D-SBPL	0.103	1.113	0.267
Age	0.171	2.241	0.026*
Peritoneal dialysis months	−0.120	−1.841	0.067
BMI	−0.036	−0.514	0.608
Total cholesterol	−0.009	−0.138	0.890
LAD	0.193	1.802	0.073
LVDD	−0.019	−0.183	0.855
LVEF	−0.075	−0.930	0.353
NT-proBNP	0.335	4.068	0.000*
Mitral valve calcification	0.132	1.926	0.056
24 h pulse pressure	−0.093	−0.930	0.354
The adjusted *R* ^2^	0.249		

Note: SE, standard error; VIF, variance inflation factor; *R* ^2^, coefficient of determination; * denotes *P* < 0.05.

**Table 6:** Multiple linear regression analysis of D-DBPL and other variables with MRA

Variables	*β*	*t*	*P*
D-DBPL	0.143	2.106	0.036*
Age	0.194	2.565	0.011*
Peritoneal dialysis months	−0.121	−1.868	0.063
BMI	−0.030	−0.427	0.670
Total cholesterol	−0.011	−0.169	0.866
LAD	0.181	1.700	0.091
LVDD	−0.028	−0.278	0.781
LVEF	−0.084	−1.041	0.299
NT-proBNP	0.329	4.049	0.000*
Mitral valve calcification	0.140	2.060	0.041*
24-h Pulse pressure	−0.047	−0.624	0.533
The adjusted *R* ^2^	0.261		

Note: SE, standard error; VIF, variance inflation factor; *R* ^2^, coefficient of determination; * denotes *P* < 0.05.

**Table 7:** Multiple linear regression analysis of N-SBPL and other variables with MRA

Variables	*β*	*t*	*P*
N-SBPL	0.036	0.475	0.635
Age	0.148	2.025	0.044*
Peritoneal dialysis months	−0.126	−1.931	0.055
BMI	−0.038	−0.526	0.600
Total cholesterol	0.005	0.072	0.943
LAD	0.192	1.779	0.077
LVDD	−0.005	−0.052	0.958
LVEF	−0.067	−0.827	0.409
NT-proBNP	0.346	4.225	0.000*
Mitral valve calcification	0.128	1.868	0.063
24 h pulse pressure	−0.038	−0.448	0.655
The adjusted *R* ^2^	0.245		

Note: SE, standard error; VIF, variance inflation factor; *R* ^2^, coefficient of determination; * denotes *P* < 0.05.

**Table 8:** Multiple linear regression of N-DBPL and other variables with MRA

Variables	*β*	*t*	*P*
N-DBPL	0.114	1.714	0.088
Age	0.168	2.286	0.023*
Peritoneal dialysis months	−0.130	−2.007	0.046*
BMI	−0.036	−0.510	0.611
Total cholesterol	−0.007	−0.111	0.912
LAD	0.181	1.685	0.094
LVDD	−0.023	−0.224	0.823
LVEF	−0.070	−0.867	0.387
NT-proBNP	0.342	4.208	0.000*
Mitral valve calcification	0.133	1.957	0.052
24 h pulse pressure	−0.045	−0.591	0.555
The adjusted *R* ^2^	0.255		

Note: SE, standard error; VIF, variance inflation factor; *R* ^2^, coefficient of determination; * denotes *P* < 0.05.

## 4 Discussion

Hypertension is one of the causes and complications of CKD [11]. Persistently high BP can exacerbate renal function deterioration. Recent research studies have determined that BP values obtained using ABPM were closely related to cardiovascular events and renal disease prognosis [8]. Consequently, 24-h ABPM provides a more comprehensive overview of a CKD patient’s BP profile [9]. The clinical relevance of BPL derived based on 24-h ABPM is increasingly recognized. Even if the average BP of patients with a high BPL is normal, it means that the heart, kidney, and other key organs and large arteries are under pressure for a longer time period, which will lead to irreversible damage to the structure and function of organs and blood vessels. Moreover, even with continuous updating and improvement of clinical diagnosis and treatment programs for CKD patients, CVD is the leading cause of death among most CKD patients [6,12]. The risk of CVD rises as the glomerular filtration rate (GFR) decreases. For every 10 mL/(min 1.73 m^2^) decrease in GFR, there is a 5% increase in CVD risk. CKD patients exhibit a CVD-related death risk that is 10–20 times higher than that of the general population [13], and CVD accounts for 50% of all-cause mortality cases among dialysis patients [3]. Elevated DBP also contributes to increased CVD risk [14]. A follow-up study by Domanski et al. on the Multi-Risk Factor Intervention Trial involving 342,815 men aged 35–57 years found that the risk of CVD death was significantly increased with higher DBP even when SBP was normal (<120 mmHg) [15]. At present, numerous studies have established a close relationship between 24-h ambulatory BPL and cardiac indicators, such as LV mass index, LV filling pressure, and LV hypertrophy, predicting the risk of CVD [16–20].

Valvular regurgitation of the heart is one of the main characteristics of CVD in ESRD patients, although there are few studies on the effect of BP on MR [5,6]. MR is the most prevalent valvular heart disease in high-income countries which can be caused by a primary structural valve abnormality (primary mitral regurgitation) or an LV disease causing incomplete closure of a structurally normal valve (secondary MR). MR in individuals without a previous history of heart disease is often considered to be a degenerative condition and the exact risk factors and preventive measures have not been identified. A longitudinal cohort study encompassing 5.5 million healthy British adults demonstrated that elevated BP increased the risk of both primary and secondary MR. Among them, for every 20 mmHg rise in SBP, the risk of MR increased by 26% [hazard ratio (HR), 1.26; confidence interval (CI) (1.23, 1.29)]. For every 10 mmHg rise in DBP, the risk of MR increased by 24% (HR, 1.24; CI (1.20, 1.28) [21]. The present study underscored hypertension as a risk factor for MR and suggested that controlling BP to reduce the risk of MR is a feasible and cost-effective measure. Therefore, the present study explored the impact of ambulatory BPL on MR in CAPD patients. The results indicated that β_2_-microglobulin level, NT-proBNP level, LAD, LVDD, and mitral valve calcification rate were higher in the high regurgitation group compared to the non-high regurgitation group. Mitral valve calcification rates were 35.29% and 13.26% in the high and non-high regurgitation groups, respectively. There was a significant difference in mitral valve calcification rate between the two groups (difference 0.22, *P* = 0.002), while hemoglobin level, LVEF, and LVFS were lower in the high regurgitation group than in the non-high regurgitation group. There was no significant difference in other clinical characteristics. The absence of a statistical difference in BPL between the two groups may be attributed to the small sample size in the high regurgitation group and a large discrepancy in sample size between the two groups. Spearman’s correlation analysis showed that there was no significant correlation between β_2_-microglobulin level, mitral valve calcification, and MRA. There was a weak positive correlation between 24h-SBPL, 24h-DBPL, D-SBPL, D-DBPL, N-DBPL, NT-proBNP level, LAD, LVDD, and MRA and a weak negative correlation between hemoglobin level, LVEF, LVFS, and MRA. Based on the correlation analysis results and the outcomes of previous studies [9,22,23], a multiple linear regression equation was constructed by incorporating BPL, age, dialysis duration, BMI, total cholesterol level, LAD, LVDD, LVEF, NT-proBNP level, mitral valve calcification, 24-h pulse pressure, and MRA. Regression analysis results showed that the effect of 24h-DBPL and D-DBPL on MRA was statistically significant (*B* = 0.010, *t* = 2.078, *P* = 0.039; *B* = 0.010, *t* = 2.106, *P* = 0.036, respectively). This suggested that BPL, especially 24h-DBPL and D-DBPL, significantly affected MRA.

At present, the mechanism of how hypertension affects the structure and function of the valve remains unclear. From a pathophysiological point of view, the mitral valve closes rapidly during the initial period of cardiac contraction due to considerable mechanical stress, followed by an increase in the pressure difference between the LV and the left atrium throughout cardiac contraction [21]. Hypertension increases the mechanical stress on the valve leaflets, which leads to endothelial cell damage and changes in the extracellular matrix, resulting in structural abnormalities, such as valve calcification, which can further lead to valve regurgitation [24]. However, many studies have shown that SBP is the most important determinant of cardiovascular events and is more closely related to MR than DBP [9,20], which is not consistent with the conclusion of the present study. The are several reasons for this observation. First, the population included in the present study analyzed PD patients with mainly elevated DBPL. Second, the mean age of the study population was 48 ± 12 years, and BP characteristics in young and middle-aged patients with hypertension included increased DBP and only slightly increased or normal SBP. This may be related to their good arterial elasticity, which can relieve vessel wall pressure without significant changes in peripheral resistance. In addition, hypertension causes an increase in left atrial diastolic pressure. During cardiac diastole, left atrial diastolic pressure will rise, which will lead to an increase in mitral valve opening pressure. This may in turn alter the flow pattern, leading to abnormal mechanical stress on the atrial surface of the leaflet and on the ventricular side of the leaflet when the valve is closed. Therefore, mechanical stress caused by elevated BP may lead to gradual changes in valve structure, resulting in severe damage to valve structure and function in the long run [21]. This demonstrates the importance and significance of controlling BP to maintain the normal structure and function of the valve.

## 5 Conclusion

Optimal BP control in CAPD patients can potentially reduce the risk of MR and subsequently decrease clinical mortality. However, the present single-center, small-sample retrospective study was not able to establish a causal relationship and its results may be prone to bias. Therefore, future research should involve multi-center, large-sample prospective studies for further validation.
